# Actions taken by female sex workers (FSWs) after condom failure in semi urban Blantyre, Malawi

**DOI:** 10.1186/s12905-020-01142-y

**Published:** 2020-12-09

**Authors:** Donatien Twizelimana, Adamson S. Muula

**Affiliations:** 1 Ekwendeni Mission Hospital, P.O. Box 19, Ekwendeni, Mzimba North, Malawi; 2 Department of Public Health, School of Public Health and Family Medicine, College of Medicine, University of Malawi, Chichiri, Private Bag 360, Blantyre, Malawi; 3 The Africa Center of Excellence in Public Health and Herbal Medicine (ACEPHEM), University of Malawi, Blantyre, Malawi

**Keywords:** Condom failure, Sexually transmitted infections, Pregnancy, Female sex workers, Blantyre, Malawi

## Abstract

**Background:**

Little is known about actions taken by female sex workers (FSWs) after male condom failure during male–female sexual intercourse. The objective of this study was to investigate the actions taken by FSWs after condom failure among FSWs in semi-urban, Blantyre in Malawi.

**Methods:**

A cross sectional, qualitative study was conducted among FSWs in Blantyre, Malawi between May and July 2019. Snowballing technique was used to recruit study participants in four purposively selected study sites. Focus group discussions and in-depth interviews were conducted by trained research assistants among 40 FSWs. Data were analyzed using thematic content analysis.

**Results:**

Study participants reported having taken different actions after condom failure. Out of 18 FSWs who experienced condom failure, 10 reported to have stopped sex immediately and changed the condom and then resumed afterwards. They reported to have douched, urinated, and/or squatted to prevent pregnancy, sexually transmitted infections (STIs) and HIV acquisition. Five study participants reported to have asked for extra pay from the client; 10 FSWs didn’t seek medical care. They thought the actions taken were enough for HIV and pregnancy prevention. Out of the 18 FSWs, only 3 stopped sexual intercourse completely and sought medical care which included post-exposure prophylaxis for HIV, STI treatment, and emergency contraceptives. Another 3 reported that they did not stop the sexual intercourse but only squatted and/or douched after sexual intercourse. The remaining 2 FSWs reported not to have stopped sexual intercourse and no any other actions were taken after the condom failure.

**Conclusion:**

We report some inadequate behaviors among FSWs after condom failure. Health programs should develop interventions and support the performance of safer sex and actions after condom failure among FSWs to prevent STIs including HIV, and unplanned pregnancies. Interpersonal, structural and policy factors hindering FSWs’ access to perform effective interventions need to be addressed.

## Background

Little is known about actions taken by female sex workers (FSWs) during male–female sex after condom failure in semi-urban Blantyre, Malawi. Condom failures (bursting and or slippage in the middle of sex) have been reported among FSWs and their male clients [1, 2]. They are several personal and public health concerns because condom failures expose female sex workers and their clients to sexually transmitted infections (STIs) and possible unintended pregnancy for the former in the absence of effective contraceptives [3, 4].

Correct condom use reduces HIV transmission in heterosexual encounters by more than 70% [5]. Proper use of condoms has also shown to prevent unintended pregnancy by 98% [3–5]. Unfortunately, condom failures are common among FSWs and they expose FSWs and their clients to STIs including HIV, and unplanned pregnancies [6–9]. The HIV prevalence among FSWs is estimated to be higher than the general population [10]. In Malawi the HIV prevalence among adult general population is around 8.8%, [11] while HIV infections prevalence among FSWs is estimated at 62.7% [12]. It is therefore important to explore actions taken by female FSWs after condom failure.

Condom failure may be arising from improper use while in some instances breakages or slippages are deliberately caused by clients [13–16]. A study conducted in Mombasa, Kenya on actions taken by FSWs after experiencing condom failure reported that FSWs had mixed options following these incidences: few singled out emergency contraceptives, several sought health care services and the majority did not know what do or where to go for assistance [1, 2]. A study in South Africa reported that about 36% of FSWs did not stop the sexual intercourse even after realizing that the condom had failed. Another 36% of the respondents stopped and put on new condom after realizing that the first one had failed. Taking no action after condom failure is similar to having condomless sex and this has potential to contribute to STI and HIV transmission and acquisition and unplanned, and possibly unwanted, pregnancies [17].

In this study we investigated FSWs’ experience with condom failure, actions taken after condom failure, awareness of consequences of condom failure, knowledge and access of pre- and post-exposure prophylaxis for HIV. Findings are expected to guide in designing policies and programs to promote FSWs’ health, their clients, partners and children.

## Methods

### Study design and population

The study was conducted between May 2019 and July 2019. In this cross sectional study, we used qualitative methods to collect data on actions taken by FSWs after condom failure in semi urban Blantyre. The study was conducted in four purposively selected townships of Chirimba, Lunzu, Kachere, Mbayani located in semi -urban Blantyre, Southern region of Malawi. Southern Malawi has the highest HIV prevalence among the general population compared to the other two regions of the country (Center and North).

The study participants were aged between 18 and 49 years, who agreed to have exchanged sex for money or goods and signed the consent. The sample had a mixture of brothel and street based women. Ten FSWs were recruited as seeds among known FSWs who usually assisted Mlambe Hospital in health promotion activities [18]. These study participants were then asked to invite their colleagues through a snowballing technique. Three female research assistants were involved in data collection: one was taking notes, the other one was facilitating the discussions and the third was sound recording the conversation. Recruitment at all four study sites continued until there was saturation and redundancy in the data being collected. In total 40 FSWs were recruited. Female sex workers who attended IDIs were randomly selected to be included in FGDs. In all, six FGDs and ten in-depth interviews were performed. On average each session of FGD had 5 study participants. Interviews and FGDs lasted between 45 and 60 min. In-depth interviews were done after FGDs. We used focus group discussion (FGD) to explore the view of the groups of FSWs, whereas in-depth interviews explored individuals’ views and experiences [19].

Data for FGDs and IDIs were collected using interview’s guide in the local language (Chichewa). The interviews explored the following topics: general, socio-economic and demographic background of the informant. There were questions on: experience with condom failure, (e.g. tell me your experience with condom rupture or slippage); and awareness of consequences of condom failure (e. g: explain in details what may be the consequences of condom rupture or slippage during sexual intercourse); explain whether you have experienced any of the consequences you mentioned. There were also questions on action taken by the study participants after condom failure (e.g.: tell us your experience after a deliberate or accidental rupture of condom during sexual intercourse, explain the use and benefits of post- exposure prophylaxis for HIV after having unprotected sex, tell us your experience and benefits of douching after unprotected sex, explain in details the benefits of squatting or passing urine after having unprotected sex). Access to condoms, contraceptives, post-exposure prophylaxis (PEP) and pre-exposure prophylaxis (PrEP) for HIV: explain your experience in accessing condoms, contraceptives, PEP, PrEP drugs from you peer FSWs, clinics, or hospitals (Additional file 1).

### Operational definitions

In this study we define female sex workers as women who sell sex in the exchange of money or goods. Condom breakage was defined as rupture of a condom during sexual intercourse. Condom slippage is getting out of a condom from a penis during sexual intercourse. Condom failure was defined as breaking, leaking, or slipping off during penetrative sexual intercourse.

### Data analysis

Data were analyzed manually using thematic content analysis relating to the objectives of the study. After data transcription, themes were identified and codes were developed. Similar themes were categorized accordingly [20].

### Ethical consideration

The study proposal was reviewed and approved by COMREC (College of Medicine Research and Ethics Committee), University of Malawi, (certificate number P.07/18/2444, dated 08-Sept-2018). The Blantyre District Council Office granted permission to conduct the study. We got clearance from the local authorities (chiefs) before the study started. Study participants were informed of the study and requested to volunteer. Written consents were obtained before the study participants were enrolled. All the information which was provided by the participants was treated with confidentiality. Cash reimbursement of Malawi Kwacha (MK) 1500.00 (approximately 2 United States Dollars at the time of data collection) was provided to all study participants as compensation for their time.

## Results

### Socio-demographic characteristics

We recruited a total of 40 study participants; 20 were between the age of 18 and 24 years. Half (20 participants) of the same study participants had steady partners. Twenty-one FSWs interviewed had attended only primary school as their highest level of academic achievement. Twenty-five (of the FSWs interviewed) were Christian. Thirty participants reported selling sex mainly at night clubs, and streets, while 10 FSWs reported to sell sex in hotels. Out of 40 study participants 18 FSWs reported incidents of condom failure and out of 18 FSWs, ten had history of condom rupture (IDIs) and eight experienced condom slippage (FGDs).

### Awareness of consequences of condom failure

Twenty five of the study participants were aware of the consequences of condom failure:

I know that if my client doesn’t use a condom properly, I can get pregnant or be infected with STI including HIV. I always try my best to avoid condom failure [Age range 25–30 years old FSW]

Many organizations come to teach us how to protect ourselves against STI including HIV. They discuss with us many issues on contraceptives. We are also taught how to help clients to use condoms properly. We know that if condoms are not used properly our clients may infect us. [FGD 1]

### Experience with condom failure

Eighteen out 40 FSWs experienced condom failure. Only five out of 18 FSWs tried to stop having sex:

One day this man took me for sex in his house. He promised me good money and we had sex. After ejaculation he did not remove his penis, after few minutes he slept. I tried to pull myself out but the condom remained. I did not go to the hospital because it was too late. The condom was removed the following day at the nearest hospital. The man convinced me that he doesn’t have HIV, ndiye basi zinatera pompho (then the issue ended like that) [Age range 20–25 years FSW].

He took me to his home we agreed on the amount to pay me. Everything was set. We started well, but later he decided to change the style. He recommended the doggy style and he was very rough than before, then the condom ruptured. He ejaculated in me. It was a deliberate action. I told him to stop but he refused. I felt very bad!!!!Why? (Asked the facilitator). Because I didn’t even know him, why did he ejaculate in me? Did you tell anybody else? (Asked the facilitator). No! Only the doctor, even my mother doesn’t know! The following day I went to the hospital, I explained everything then I was treated with injections and pills. They tested my blood then they told me that I am HIV positive, I am not sure if I got the virus from the previous men I had sex with or this last client. [Age range 20–25 years old FSW].

You don’t know men…they puncture condoms with finger nails deliberately, so that they can enjoy sex without condom [Age range 20–25 years old FSW]

Other women’s focus was only on the amount of money paid by their clients after sexual intercourse. Condom failure was not an issue.

While having sexual intercourse we realized that the condom has ruptured. I told the man to come out and ejaculate outside but he refused and he said that he will give me MK 20,000 Kwacha, I accepted then he continued until he finished. That day I made enough money to feed my children. [Age range 30–35 years old FSW]

What we want is money. Most of our friends (FSWs) are on ARVs, we know each other. We are already sick. Some men refuse to use condoms. We accept them. We charge them for condomless sex. (FGD 4).

I am a sex worker and I know already the risks, I can catch HIV and other sexual transmitted infections. As long as he pays me well, he can continue enjoying the sex even after condom rupture. [Age range 25–30 years old FSW]

### Action taken by FSWs after condom failure

Study participants reported having taken different actions after condom failure. Out of 18 FSWs who experienced condom failure, ten reported to have stopped sex immediately, changed the condom before resuming intercourse. They reported to have douched, urinated, and/or squatted to prevent pregnancy, sexually transmitted infections (STIs) and HIV acquisition. Five asked for extra pay from the client; 10 FSWs didn’t seek medical care. They thought the actions taken were enough for HIV and pregnancy prevention. Out of the 18 FSWs, only 3 stopped sexual intercourse completely but sought medical care which included post-exposure prophylaxis (PEP) for HIV, STI treatment, and contraceptives. The other 3 reported that they did not stop the sexual intercourse but only squatted and/or douched after sexual intercourse. The remaining 2 FSWs reported not to have stopped sexual intercourse and no any other actions were taken after the condom failure. Due to stigma and discrimination FSWs often were not willing to share their experience on condom failure with anybody else.

Ine zinandichitikira, kondomu inaphulika (I experienced condom failure) I shared my experience with one closest friend who is also a FSW. One week later I heard the same story from many FSWs here at Kachele. It is not good to share the experience with anybody else. Even if you discuss about it with your client it is just a waste of time. He can shout at you. To go to the hospital, manyazi!(I failed to go to the hospital because I was feeling shy to explain the incident to the health care provider) Only God knows. [Age range 15–20 years old FSW]

After urinating, squatting, or douching, sperm and all viruses come out. But HIV….. I am not sure, but pregnancy no!! Believe me! But the men must pay extra money after condom rupture [Age range 30–35 years old FSW]

We do douching or squatting or urinate always after unprotected sex. We use water or beer (when water is not available). After douching you become smart and the vagina outlet becomes narrow while you are waiting for the next client. It really makes you smart but beer’s smell is not good but in the absence of water you can use it. When douching you remove all diseases and sperm. [FGD 2]

It depends on where you are. I met one client who took me to………. Hotel. We had sex the whole night and during the process the condom ruptured. I went to the toilet and had a bath and the man changed the condom and then we resumed the sexual intercourse. There was no need to go to the hospital. It was too late and the hospital was very far. [Age range 15–20 years old FSW]

I went to the hospital after the condom ruptured while having sex with one of my clients. The doctor tested my blood for HIV. Fortunately I was negative. I was given ARVs for HIV prophylaxis. I was also given some contraceptives, one injection and some tablets. I felt very uncomfortable with the injection and the ARVs. It is good to avoid unplanned pregnancies and HIV but we feel very uncomfortable to explain the circumstances of condom rupture. [Age range 20–25 years old FSWs]

### Access to contraceptives, pre- and post-exposure prophylaxis (PrEP &PEP) of HIV

Nineteen study participants reported to have limited knowledge on ARVs. They have challenges in accessing them. They reported to be easily accessed on different sites where sex work is done.

We heard that there are some ARVs which protect people against HIV. Most of us are not aware of them. [FGD 3]

Accessing those drugs it’s not easy, they are very scarce in Government hospitals. Sometimes you can spend the whole day there at the health center then you go back in the evening without drugs. We can’t afford to settle the bills in private hospitals. [FGD 4]

We can’t get those drugs (for HIV, PEP&PrEP) from our peer FSWs because they don’t keep secrets, they even tell our clients and other FSWs about our status. Once our clients are aware that we are sick, they stop coming to us, so we lose business. [FGD 5]

Many NGO bring condoms, drugs, vaginal lubricants and some contraceptives. They are very helpful because most of the times they find us either in our homes, night clubs or by the road. No need to go to the hospital. [FGD 6]

## Discussion

Many studies conducted among FSWs and on condom use have focused more on FSWs perception and inconsistent use of condom; however, fewer studies have reported on experiences with action taken by the female sex workers after condom failure. The analyses presented here provide critical evidence from the FSWs in different scenarios.

Findings show that many study participants were aware of the consequences of condom failure. They mentioned unintended pregnancies and sexually transmitted infections including HIV. Knowledge of those consequences did not decrease the risk-taking behaviors among some study participants. It is also reported that 5 out 18 FSWs who had condom failure in this study asked for higher pay. This is consistent with study findings from other setting [21]. Many FSWs in Malawi live in poverty, they have many socio-economic challenges which make them likely to accept increased pay for unprotected sex which may results in getting pregnant, HIV, or both [22, 23]. FSWs failure to negotiate condom use and continuing with condomless sex to earn more money are barriers to successful condom use and result in loss of situational control [24–29]. Empowering FSWs to improve their socio-economic status may be helpful in addressing FSWs financial challenges as well as risk-taking behaviors [25–29].

Female sex workers who could be living with HIV and experienced condom failure also continued with condomless sex. In one of the FGDs, FSWs reported that they are more interested on the amount of money paid by clients. In this study we found that 18 of the study participants had limited knowledge on ARVs, specifically PEP and PrEP. Twenty one FSWs interviewed had attended only primary school as their highest level of academic achievement. Limited education, active sexual behavior and limited knowledge about HIV prevention and transmission characterize most of the study participants. Knowledge, attitude, and practice model can be used as an intervention to reduce risk-taking behaviors among FSWs. According to this model, an individual’s knowledge about a disease positively affects their attitude toward disease prevention and may reduce risk-taking behavior [30–32]

Female sex workers often blamed their clients as intentionally causing condom failure. Female sex workers reported that men puncture, remove, or cause the condom to slip. This is consistent with other studies done elsewhere [33]. However in our study we looked at all the circumstances of condom failure, we found that not all of them were intentionally caused by FSWs’ male partners. For instance, one FSW reported that after sexual intercourse, there was condom slippage when the partner fall asleep. The condom was removed from her vagina the following day. This reflects improper use of condom. There is a need for special training programs on condom use among FSWs and their partners. These programs have been reported to be effective in reducing condom failure and HIV prevalence in some FSWs communities [33–36].

The study participants reported that often times, efforts to stop men to continue sex after condom failure failed. Our study findings suggest that men used physical force to continue having sex despite FSWs’ request to stop the act due to condom failure. This reflects that FSWs became more submissive to the dominants partners to avoid the exacerbation of violence. Despite their knowledge regarding the risk of STIs transmission, and unplanned pregnancy, FSWs were not able to negotiate for condom use after noting the failure. FSWs fail to negotiate for condom use not only on the account of being women but also due to being in unequal financial level with male partners [37, 38]. Their clients are their source of income and sometimes the more they pay, the more they dictate the way they want to have sex. This highlights the need for interventions targeting FSWs on how to negotiate condom use with clients. The interventions should empower FSWs with the skills, ability, and power of refusing to have sex if the client is not using a condom, refusing to have sex for more money if a client is not willing to use a condom, and convincing an unwilling client to use a condom [39, 40]. There is wide range of reasons given by FSWs clients for not stopping sexual act after condom failure. Men may not want to compromise the sexual enjoyment and also they want to enjoy the sex they have paid for.

After condom failure only 3 out 18 FSWs who experienced condom failure decided to seek medical care. Others did not take any further actions other than replacing the first condom, squatting, urinating and vaginal douche. They did not know that further actions (such as PEP) may reduce the like hood of STI including HIV. Further, study participants were aware of health seeking actions but did not go to the health facilities due to stigma and fear of discrimination. They did not feel comfortable to explain to the health care provider the circumstances which resulted in condom failure. In the health care system there is a need to provide stigma-free emergency contraception and HIV and STI post-exposure prophylaxis in the aftermath of a condom failure [41].

As indicated earlier FSWs believed that urinating, squatting, washing the vagina with water or beer following condom failure would protect them from STIs including HIV, and getting pregnant. Only 3 out of 18 FSWs were aware of post exposure prophylaxis (PEP) for HIV, they went to the health facility for HIV testing and prophylactic care after condom failure. This is consistent with some other studies which reported low level of awareness of PEP and PrEP among FSWs [42]. Specific health literacy programs on post exposure prophylaxis (PEP) and pre exposure prophylaxis (PrEP) for HIV are imperative in this community to reduce the risk of HIV acquisition. It is important to provide services close to FSWs to achieve healthy future to them, their clients and children.

Our study findings suggest that there are multilevel challenges to contraceptives, PrEP and PEP access. Interpersonal factors such as fear of stigma and discrimination by other sex workers, family and society are barriers to the access of health care in the aftermath of condom failure among the study participants. In addition, structural and policy related factors were found in our study. There were privacy concerns and fear of breach of confidentiality. Based on our study findings we suggest that health care providers should provide contraceptives, PEP, and PrEP services among FSWs in outreach services where they are easily found: bars, night clubs and brothels. This approach not only will increase the uptake of PEP, PrEP services, and contraceptives, it will also remove transport cost to the static health care facilities and other inconveniences such as long waiting hours at the health facility [43].

### Limitations of the study

We used snowball method to sample our study participants. In this process there is no possibility of generalizing the research findings to the general population identified because the process itself has high possibility of producing biased samples and results [44]. In snowballing method participants have a tendency of referring their friends and contacts, this may result in a sample of over representation of individuals who share similar characteristics. [45]. Sex work is a sensitive issue, as such there is potential for social desirability bias. Further, our data were obtained from female sex workers and not triangulated with clients’ narratives. The sample size was relatively small, and with the study sample drawn from the population of one city in Malawi, this may limit the generalizability of results to other contexts.

## Conclusion

This study reported on experiences and identified the actions taken by FSWs when a male condom fails during sexual act with a male client. A similar study may be required when a female condom is used. The unveiled findings such as continuing the sexual intercourse without a new condom, doing nothing at all could expose the sex worker to getting pregnant, contracting STIs including HIV. There is a need to improve the health literacy and use of emergency contraceptives, pre- and post-exposure prophylaxis for HIV, and STI treatment. There is a need for interventions to mitigate the risk of HIV, STIs, and pregnancy in the aftermath of a condom failure. There is a need to address misconceptions related to health illiteracy among FSWs.

## Supplementary Information

**Additional file 1**. Data collection tool for qualitative data (IDI&FGDs).

## Data Availability

The datasets used and/or analyzed during the current study are available from the corresponding author on reasonable request.

## Abbreviations

ACEPHEM: Africa Center of Excellence in Public Health and Herbal Medicine
ARVs: Antiretroviral drugs
COMREC: College of Medicine Research and Ethics Committee
FGDs: Focus group discussions
FSWs: Female sex workers
HIV: Human immunodeficiency virus
MK: Malawi kwacha
NGO: Non-governmental organisation
PEP: Post-exposure prophylaxis for HIV using antiretroviral medicines
PMTCT: Prevention of mother to child transmission
PrEP: Pre-exposure prophylaxis for HIV using antiretroviral medicines
STIs: Sexually transmitted infections
