# Supplementary material for: Actions taken by female sex workers (FSWs) after condom failure in semi urban Blantyre, Malawi
Source: BMC Womens Health. 2020 Dec 9;20:273. doi: 10.1186/s12905-020-01142-y (PMC7727183; doi:10.1186/s12905-020-01142-y)
Supplement: Supplementary file 1 — Additional file 1. Data collection tool for qualitative data (IDI&FGDs). [file 12905_2020_1142_MOESM1_ESM.docx]

**ACTIONS TAKEN BY FSWs AFTER CONDOM FAILURE IN SEMI URBAN BLANTYRE.**

**DATA COLLECTION TOOL FOR QUALITATIVE DATA (IDI&FGDs)**

**ENGLISH VERSION**

1. **EXPERIENCE WITH CONDOM USE**
2. How comfortable are you when using male condom? Explain in details
3. How consistently do you use male condom? Explain in details.
4. Explain in details the willingness of your clients to use a condom?
5. What reasons do men have when they refuse to use condoms? What do you do after your clients have refused to use a condom? Explain in details
6. Explain your experience with condomless sex in exchange with high pay. Explain in details the action taken after the encounter.
7. **EXPERIENCE WITH CONDOM FAILURE**
8. Tell me your experience with condom rupture or slippage? Explain in details
9. Let me know whether you have experienced a situation where your client told you that he has put on condom while in reality he did not. Explain how it happened. What did you do after sexual intercourse?
10. Why do you think men continue to have sex after condom rupture or slippage?
11. In terms of trying to stop your partner to discontinue having sex after condom failure, how likely is this going to happen?
12. **AWARENESS OF CONSEQUENCES OF CONDOM FAILURE**
13. Explain in details what may be the consequences of condom rupture or slippage during sexual intercourse. Tell us whether you have been the victim of any of the consequences mentioned.
14. **ACTION TAKEN BY FSWs AFTER CONDOM FAILURE**
15. Tell us your experience after a deliberate or accidental rupture of condom during sexual intercourse.
16. Explain whether and how you shared the condom rupture’s experience with your friends, relatives, husband or partners.
17. Explain whether it’s relevant to discuss about the same condom rupture with your client.
18. Tell us your experience and benefits of douching after unprotected sex.
19. Explain in details the benefits of squatting or passing urine after having unprotected sex.
20. Explain in details the advantages and disadvantages of going to a health care provider after condom rupture.
21. Explain in details the use and benefits of emergency contraceptives. Explain the use and benefits of post exposure prophylaxis for HIV after having unprotected sex
22. **ACCESS TO HEALTH CARE PROVIDER**
23. Explain your experience in accessing condoms?
24. Explain your experience in accessing contraceptives?
25. Explain your experience in accessing ARVs including PEP, PreP drugs from you peer FSWs?
26. Explain your experience in accessing ARVs including PEP, PreP drugs from clinics, or hospitals.
